# Continuous renal replacement therapy (CVVHD) for acute kidney injury in critical care: incidence and outcome across South West Wales

**DOI:** 10.1186/cc13581

**Published:** 2014-03-17

**Authors:** K Brown, M Challis, A Mikhail

**Affiliations:** 1 Morriston Hospital, Swansea, UK

## Introduction

Renal replacement therapy in critical care is associated with increased mortality. It is not known for which patients RRT confers the most benefit, or who will recover function and remain dialysis independent on a long-term basis.

## Methods

All ICU patients receiving CVVHD (diffusive haemodialysis) for AKI were analysed in a retrospective cohort study of mixed non/surgical patients at a tertiary renal centre over a 2-year period (December 2011 to November 2013). Children <18 years, patients on intermittent haemodialysis or patients requiring plasma exchange were excluded from analysis.

## Results

A total of 2,297 patients were admitted to the ICU, of which 14% (319) required CRRT. Thirteen patients were excluded from analysis; *n *= 306, of which 58% were male. Causes of AKI included sepsis (37%), surgery (13%, of which 82% were emergency procedures) and vascular events (9%). Mean values for patients requiring RRT versus all ICU patients: age (65 vs. 62.5 years), APACHE II score (21.2 vs. 14.8), length of stay (13 vs. 8.2 days). Forty-seven per cent of patients received incident dialysis <24 hours of admission, with mean flow rates of 31 ml/kg/hour (2 l) in 39%. Mortality at hospital discharge was 56% for RRT versus 20% in all ICU patients admitted over the same time period.

## Conclusion

The reported incidence of AKI in critical care ranges from 20 to 50%, with the highest rates seen in sepsis [1]. Utilisation of CRRT for AKI is higher at our centre than the described 5% [2], potentially due to close collaboration between critical care and nephrology [3] or relatively lenient ICU admission criteria. Increasing mortality was seen with age, APACHE II score and delay in initiation of RRT. Prospective analysis is required to look at determining biomarkers for AKI and risk factors for mortality; dynamic monitoring of haemodynamic responders (↑MAP, ↓vasopressor requirement <24 hours), percentage creatinine decrease [4], severity-of-illness scores and urine output. Results of current RCTs are awaited, which may provide more information on mode of clearance, flow rates and early versus standard initiation of RRT to more accurately prognose patients' outcomes.
